# A Study of the Relationship between the Polymorphism and Mutation of rs682429 and rs3781590 in the LRP5 Gene and Bone Metabolism in Postmenopausal Type 2 Diabetic Women in Xinjiang

**DOI:** 10.1155/2020/3071217

**Published:** 2020-04-28

**Authors:** Jun Li, SiYuan Li, HuiRong Zhao, JiaJia Li, Shuang Wang, YanQiu Shi

**Affiliations:** ^1^Department of Endocrinology and Metabolism, The First Affiliated Hospital, Shihezi University School of Medicine, Shihezi, Xinjiang 832002, China; ^2^Medical College, Shihezi University, Shihezi 832002, China; ^3^Department of Endocrinology and Metabolism, The Second People's Hospital of Nanyang, Nanyang, Henan 473000, China; ^4^Department of Endocrinology and Metabolism, The Central Hospital of Yangpu District in Shanghai, Shanghai 200000, China; ^5^Department of Cardiovascular Medicine, Xiaoshan Hospital of Hangzhou in Zhejiang, Hangzhou, Zhejiang 310000, China

## Abstract

**Objective:**

To explore the expression of the polymorphism and mutation of rs682429 and rs3781590 in the low-density lipoprotein receptor-related protein 5 (LRP5) genotype and to analyse the relationship of bone mineral density (BMD) and bone metabolism markers in postmenopausal women with type-2 diabetes mellitus (T2DM) in Xinjiang, China, to provide a basis for prevention and treatment of the disease.

**Methods:**

A total of 136 postmenopausal women were included in the study. According to the results of an oral glucose tolerance test (OGTT) and dual-energy X-ray (DEXA) determination of BMD, the study subjects were divided into 4 groups: group A: normal OGTT+normal bone mass group; group B: normal OGTT+osteoporotic (OP) group; group C: T2DM+normal bone mass group; group D: T2DM+osteoporotic (OP) group. Calcium (Ca), phosphorus (P), alkaline phosphatase (ALP), and clinical biochemical data were determined; haemoglobin A1c (HbA1c) was measured by HPLC; BMD of the femoral neck, hip, and lumbar spine (L1-4) was measured by dual-energy X-ray (DEXA); and the rs682429 and rs3781590 polymorphisms of the LRP5 gene were detected by time-of-flight mass spectrometry (TOF MS).

**Results:**

(1) The rs682429 polymorphism of the LRP5 genotype distribution was statistically significant (*P* < 0.05) in group B compared with group A. (2) The triglycerides (TG) of women with the CT/TT genotype (mutant type) were higher than those of women with the CC genotype (wild type) (2.37 ± 1.30 vs. 1.52 ± 0.83, *P* < 0.05) at the rs3781590 site of the LRP5 gene in group D. (3) Multiple linear regression analysis showed that TG (*β* = 0.034, *P* < 0.05) and body mass index (BMI) (*β* = 0.013, *P* < 0.05) were the influencing factors of BMD (L1-4) in T2DM patients. TG (*β* = 0.022, *P* < 0.05), BMI (*β* = 0.009, *P* < 0.05), and duration of menopause (*β* = 0.005, *P* < 0.05) were the influencing factors of BMD (hip).

**Conclusion:**

(1) The rs682429 polymorphism site in the LRP5 gene may be involved in bone metabolism in postmenopausal women from Xinjiang. (2) The rs3781590 mutation in the LRP5 gene from these subjects may be involved in lipid metabolism. (3) Among postmenopausal women with type 2 diabetes mellitus and bone mass abnormality in the Xinjiang Shihezi area, high BMI and TG are protective factors against increased BMD. Duration of menopause is a risk factor for increased BMD.

## 1. Introduction

Type 2 diabetes mellitus (T2DM) is a clinical syndrome that mainly presents as disordered glucose metabolism and is characterized by chronic hyperglycaemia. Genetic susceptibility plays an important role in the development of this disease [1]. Osteoporosis (OP) is a systemic bone disease, which is characterized by decreased BMD and increased risk for fracture [2]. Postmenopausal osteoporosis, the most common OP, is usually associated with oestrogen deficiency [3]. LRP5, a member of the low-density lipoprotein cholesterol (LDL-C) receptor family, is the transmembrane receptor of the Wnt protein in this signalling pathway [4]. Studies have shown that LRP5 has a large influence on the Wnt signalling pathway and regulates the growth and the differentiation of osteoblasts by controlling BMD and bone metabolism [5]. Furthermore, LRP5 also plays an important role in the metabolism of blood lipids and blood glucose. Various studies have shown that LRP5 is a potential susceptibility gene for T2DM [6]. Therefore, the purpose of this study was to observe the relationship between polymorphism and mutation of the LRP5 gene and bone metabolism in postmenopausal T2DM in Xinjiang.

## 2. Materials and Methods

### 2.1. Participants

We collected 136 cases of natural postmenopausal women from the First Affiliated Hospital of Medicine School in Xinjiang, China, from November 2018 to April 2019. According to the results detected by an oral glucose tolerance test (OGTT) and dual-energy X-ray (DEXA) for BMD, the subjects were divided into four groups: normal glucose tolerance and bone mass group (group A), which included 26 patients; normal glucose tolerance and abnormal bone mass group (group B), including 28 patients; T2DM group with normal bone mass (group C), including 27 cases; and T2DM group with bone mass abnormality (group D), including 55 cases. All the patients included in this study signed the written informed consent. This study was approved by the ethics committee.

### 2.2. Procedures

To calculate the body mass index (BMI) and waist-to-hip ratio (WHR), each subject's height, weight, waist circumference, and hip circumference were determined. The subjects were tested for fasting plasma glucose (FPG), the 2-hour blood glucose after oral glucose tolerance test (2-h OGTT PG), haemoglobin A1c (HbA1c), high-density lipoprotein cholesterol (HDL-C), low-density lipoprotein cholesterol (LDL-C), triglycerides (TG), calcium (Ca), phosphorus (P), and alkaline phosphatase (ALP). Ca, P, ALP, FPG, 2-h OGTT PG, HDL-C, LDL-C, and TG were measured by a Roche automatic biochemical analyser (Modular DPP-H7600), and HbA1c was tested by high-performance liquid chromatography (HPLC). The dual-energy X-ray absorptiometry (DEXA) method was used to determine the bone mineral density of the lumbar spine and the femur. The rs682429 and rs3781590 polymorphism sites in the LRP5 genotype were detected by time-of-flight mass spectrometry (TOF-MS). T2DM was diagnosed according to the 1999 WHO criteria. BMD was diagnosed according to the 1994 WHO criteria.

## 3. Statistics

The data were analysed by using SPSS 20.0 software. At the rs682429 and rs381590 loci, polymorphisms and allele frequency of the LRP5 genotype were determined. The chi-square test was used to determine the compatibility of observed genotype frequencies with that of Hardy-Weinberg equilibrium. The clinical variables were presented as the mean ± standard deviation (SD, −*x* ± *s*). If the baseline data were homogeneous in distribution, the analysis was done by single-factor ANOVA. However, if baseline data were not homogeneous in distribution, they were analysed using covariance analysis (ANCOVA). Multivariate linear regression analysis was used to analyse the factors affecting bone density. A *P* value <0.05 was considered statistically significant.

## 4. Results

### 4.1. Comparison of Baseline Data among Groups

This text presents the expression data in Table 1. Age and period of menopause were significantly higher in group B and group D than in group A (*P* < 0.05). Baseline data were not homogenous. ANCOVA analysis was used to exclude the effects of age and menopause duration on the outcome.

### 4.2. Covariance Analysis of Biochemical Indices among the Study Groups after ANCOVA Analysis

The results of comparison among the groups showed that compared with those of group A, levels of FPG and HbA1c% of group C and group D were higher (*P* < 0.01), and the TG level of group D was lower (*P* < 0.05) (Table 2).

### 4.3. Analysis of LRP5 Gene Polymorphism

This study showed that the LRP5 rs682429 genotype distribution was in line with Hardy-Weinberg equilibrium (*P* > 0.05) in group A, group B, group C, and group D. As shown in Table 3, the rs682429 polymorphism of the LRP5 gene distribution was statistically significant (*P* < 0.05) in group B compared with group A.

### 4.4. Comparison of rs3781590 and rs3781590 in the LRP5 Genotype and BMD, Bone Metabolism, Glucose Metabolism, and Lipid Metabolism

The biochemical indexes were not statistically significant between the wild-type and mutant-type genotypes of rs682429 and rs3781590 in the LRP5 gene (*P* > 0.05) in groups B and C. The BMD (femoral neck) of the CC genotype (wild type) of rs3781590 in the LRP5 gene was higher than that of the CT/TT genotype (mutant type) (0.92 ± 0.12 VS. 0.80 ± 0.05, *P* = 0.024) in group A, but the difference was not statistically significant after age, menopause, and BMI were adjusted (*P* = 0.220). The TG of the CT/TT genotype (mutant type) was higher than that of the CC genotype (wild type) (2.37 ± 1.30 VS. 1.52 ± 0.83, *P* < 0.05) at the site of rs3781590 in the LRP5 gene in group D (Table 4).

### 4.5. Multiple Linear Regression Analysis

Multiple linear regression was used to analyse the influencing factors of BMD in T2DM patients. The dependent variables were BMD (L1-4), BMD (hip joint), and BMD (femoral neck). Thirteen independent variables were included: age (*X*1), menopausal years (*X*2), BMI (*X*3), WHR (*X*4), FPG (*X*5), HbA1c (*X*6), ALP (*X*7), TG (*X*8), HDL-C (*X*9), LDL-C (*X*10), Ca (*X*11), P (*X*12), and genotype (*X*13). The results showed that TG and BMI were positively correlated with the level of BMD (L1-4) and BMD (hip joint) but negatively correlated with menopausal years (Table 5).

## 5. Discussion

Following the rapid development of the economy, the lifestyle and eating habits of people also have changed. High incidence and high morbidity of OP bring a heavy burden to families [7]. In this study, the BMD of group C and group D was lower than that of group A. Although the difference between groups was not statistically significant (*P* > 0.05), it reflected the observation that postmenopausal women with T2DM were more likely to have OP, which was consistent with the results of Chen et al. [8]. Therefore, postmenopausal women with T2DM should pay attention to screening and early prevention of OP.

It is unknown whether T2DM causes a decrease in BMD. However, more studies have shown that T2DM may lead to increased bone fragility [9] and promote the occurrence and development of T2DM combined with OP [10]. The pathogenesis of T2DM combined with OP is multifactorial, often affected by genetic and environmental factors. Polygenes have been found to be associated with T2DM combined with OP. Our study found that the genotype and allele frequency distribution of rs682429 and rs3781590 in the LRP5 gene were consistent with Hardy-Weinberg equilibrium. The high allele frequency of rs3781590 in the LRP5 gene was 0.82, which was similar to the gene frequency distribution of populations in Korea [11], Japan [12], Shanghai [13], and Australia [14]. This indicated that there might be racial differences in the allele frequency distribution of rs3781590 and rs682429 in the LRP5 gene. Our study found that the rs682429 polymorphism in the LRP5 genotype may be involved in bone metabolism in postmenopausal women from Xinjiang, which was consistent with the research results presented above. The results of multiple linear regression analysis showed that TG and BMI were protective factors, and menopausal years was a risk factor for increased BMD.

Studies have shown that polymorphism of the LRP5 gene is associated with an increased LDL-C level, increased BMI, and obesity and presents a linkage imbalance [15]. In this study population, the TG of the CT/TT genotype (mutant type) was higher than that of the CC genotype (wild type) of rs3781590 in the LRP5 gene in group D, suggesting that the mutation of rs3781590 in the LRP5 gene might be involved in lipid metabolism.

To summarize, the LRP5 gene was used as the action target to explore the relationship between the polymorphism and mutation of rs682429 and rs3781590 of the LRP5 gene and bone metabolism in postmenopausal women with T2DM. The results suggest that the rs682429 polymorphism in the LRP5 genotype may be involved in bone metabolism in postmenopausal women from Xinjiang. The rs3781590 mutation in the LRP5 gene may be involved in lipid metabolism. This study provides a basis for the prevention and treatment of this disease.

## Figures and Tables

**Table 1:** Comparison of baseline data among groups (−*x* ± *s*).

Variable	Group A	Group B	Group C	Group D
Age/years	65.62 ± 8.18	69.71 ± 7.26∗	66.52 ± 7.54	70.44 ± 6.44∗∗
Menopausal period	15.73 ± 7.94	19.71 ± 7.26∗	16.70 ± 7.08	20.44 ± 6.44∗∗
BMI (kg/m^2^)	25.66 ± 3.02	25.68 ± 4.72	27.08 ± 3.99	25.68 ± 3.46
WHR	0.87 ± 0.18	0.88 ± 0.08	0.90 ± 0.05	0.91 ± 0.06

VS group A, ∗*P* < 0.05, ∗∗*P* < 0.01. BMI: body mass index; WHR: waist-hip ratio.

**Table 2:** Comparison of biochemical indices among groups after ANCOVA analysis (−*x* ± *s*).

Variable	Group A	Group B	Group C	Group D
FPG (mmol/L)	5.18 ± 2.13	5.15 ± 2.11	7.60 ± 2.13∗∗	7.50 ± 2.13∗∗
HbA1c %	5.92 ± 1.06	5.98 ± 1.04	7.19 ± 1.05∗∗	7.50 ± 1.05∗∗
TG (mmol/L)	2.50 ± 1.52	1.51 ± 1.50∗	2.26 ± 1.52	1.70 ± 1.52∗
HDL-C (mmol/L)	1.35 ± 0.33	1.31 ± 0.33	1.18 ± 0.33	1.21 ± 0.33
LDL-C (mmol/L)	3.06 ± 1.01	3.17 ± 0.99	3.18 ± 1.01	3.47 ± 1.01
Ca (mmol/L)	2.27 ± 0.14	2.29 ± 0.14	2.25 ± 0.14	2.28 ± 0.14
P (mmol/L)	1.09 ± 0.79	1.07 ± 0.78	1.11 ± 0.79	1.24 ± 0.79
ALP (U/L)	80.26 ± 21.26	82.13 ± 20.99	72.37 ± 21.18	73.79 ± 21.21
BMD (L1-4)	1.189 ± 0.15	0.845 ± 0.14∗∗	1.217 ± 0.15	0.911 ± 0.15∗∗
(g/cm^2^)				
BMD (femoral neck) (g/cm^2^)	0.882 ± 0.18	0.715 ± 0.19∗∗	0.801 ± 0.19	0.754 ± 0.19∗∗
BMD (hip) (g/cm^2^)	0.963 ± 0.11	0.778 ± 0.11∗∗	0.944 ± 0.11	0.809 ± 0.11∗∗

VS group A, ∗*p* < 0.05, ∗∗*p* < 0.01. FPG: fasting plasma glucose; HbA1C: glycosylated haemoglobin; TG: triglyceride; HDL-C: high-density lipoprotein cholesterol; LDL-C: low-density lipoprotein cholesterol; ALP: alkaline phosphatase; BMD: bone mineral density.

**Table 3:** Comparison of genotype frequencies of rs682429 in LRP5 among groups.

Groups	Case	GG *n* (%)	GA *n* (%)	AA *n* (%)	*P* value
A	25	13 (52.0)	10 (40.0)	2 (8.0)	
B	28	5 (17.9)	15 (53.6)	8 (28.6)	0.018∗
C	26	6 (23.1)	17 (65.4)	3 (11.5)	0.101
D	55	17 (30.9)	27 (49.1)	11 (20.0)	0.144

VS group A, ∗*P* < 0.05.

**Table 4:** Comparison of biochemical markers of different genotypes of rs3781590 in the LRP5 gene in group D (−*x* ± *s*).

Variables	CC	CT/TT
FPG (mmol/L)	7.45 ± 1.95	7.26 ± 1.51
HbA1c(%)	7.41 ± 1.07	7.78 ± 1.39
TG (mmol/L)	1.52 ± 0.83	2.37 ± 1.30∗
HDL-C (mmol/L)	1.23 ± 0.31	1.16 ± 0.25
LDL-C (mmol/L)	3.40 ± 1.03	3.60 ± 1.12
Ca (mmol/L)	2.27 ± 0.11	2.28 ± 0.08
P (mmol/L)	1.29 ± 1.34	1.08 ± 0.09
ALP (U/L)	73.52 ± 20.28	73.18 ± 31.36
BMD (L1-4)	0.90 ± 0.12	0.94 ± 0.09
(g/cm^2^)		
BMD (femoral neck) (g/cm^2^)	0.74 ± 0.11	0.75 ± 0.11
BMD (hip) (g/cm^2^)	0.79 ± 0.11	0.83 ± 0.15

∗*P* < 0.05. FPG: fasting plasma glucose; HbA1C: glycosylated haemoglobin; TG: triglyceride; HDL-C: high-density lipoprotein cholesterol; LDL-C: low-density lipoprotein cholesterol; ALP: alkaline phosphatase; BMD: bone mineral density.

**Table 5:** Multivariate linear stepwise regression analysis of the influencing factors of BMD in T2DM patients.

BMD	Variable	*β*	SE	*t* value	*P* value
L1-4					
	TG	0.034	0.014	2.456	0.016∗
	BMI	0.013	0.006	2.288	0.025∗
Hip joint	TG	0.022	0.009	2.450	0.017∗
	BMI	0.009	0.004	2.336	0.022∗
	Menopausal period	-0.005	0.002	-2.225	0.029∗

∗*P* < 0.05. TG: triglyceride; BMI: body mass index.

## Data Availability

All data, models, or code used during the study are available from the corresponding author by request.
